# Adolescents’ unhealthy snacking behaviour during the school journey and the association with transport modes and food outlets along the school route

**DOI:** 10.1177/17579139241257091

**Published:** 2024-08-13

**Authors:** ML Situmorang, S Mandic, M Smith, M Keall, N Donnellan, KJ Coppell

**Affiliations:** Department of Medicine, University of Otago, New Zealand; Centre for Sustainability, University of Otago, New Zealand; Centre for Sustainability, University of Otago, New Zealand; AGILE Research Ltd., New Zealand; Faculty of Health and Environmental Sciences, School of Sport and Recreation, Auckland University of Technology, New Zealand; School of Nursing, University of Auckland, New Zealand; Department of Public Health, University of Otago Wellington, New Zealand; School of Nursing, University of Auckland, New Zealand; Department of Medicine, University of Otago Wellington, Wellington South 6242, New Zealand

**Keywords:** adolescents, unhealthy snacks, school transport modes, school route, food outlets, neighbourhood deprivation

## Abstract

**Aims:**

Active transport to and from school provides an opportunity for adolescents to engage in physical activity, but travelling through an obesogenic environment may have unintended consequences on their snacking behaviour. This study aimed to: (1) identify whether adolescents’ unhealthy snacking behaviour was associated with school transport modes and food outlets on their estimated school route and (2) explore whether food outlet density on the school route differed between school transport modes.

**Methods:**

Adolescents (*n* = 406; aged 15.1 ± 1.4 years; 50.7% boys; 63.5% New Zealand European) from all 12 secondary schools in Dunedin city, Aotearoa New Zealand, completed an online survey. School transport modes (active, motorised or mixed) and unhealthy snacking data were collected. Food outlet data were collected using Google Places Application Programming Interface (API). Home-to-school route and distance were estimated using geographical information system (GIS) analysis based on a walkable road network. Data were analysed using the chi-square test and logistic regression.

**Results:**

Overall, 26.4% of adolescents reported purchasing and consuming unhealthy snacks or soft drinks on the way to school and 41.4% from school. The odds of unhealthy snacking during the school journey was higher among mixed transport users than active transport users on the way to (odds ratio (OR) = 2.73, 95% confidence interval (CI) = 1.39–5.36) and from school (OR = 2.68, 95% CI = 1.40–5.13). No differences were observed by food outlet type. There were no food outlets on the estimated school route for 44.8% of adolescents. The presence of more than one food outlet per kilometre of the estimated school route differed between active (38.7%), motorised (42.6%) and mixed transport users (46.4%; *p* = 0.006).

**Conclusion:**

School transport modes were significantly associated with adolescents’ unhealthy snacking behaviour and food outlets on their school journey. Policy measures which minimise exposure to unhealthy food outlets may reduce unhealthy snacking among adolescents.

## Introduction

Obesity is a risk factor for many increasingly common non-communicable diseases such as type 2 diabetes, metabolic dysfunction-associated fatty liver disease, osteoarthritis and some cancers such as breast and colon cancer.^1^ Globally, obesity among adolescents increased 10-fold from 5 to 50 million girls and 6 to 74 million boys between 1975 and 2016.^2^ In Aotearoa New Zealand (New Zealand), 38% of young adolescents aged 10–14 years and 46% of those aged 15–24 years were classified as overweight or obese in 2022/23.^3^ While the development of obesity in adolescents is complex, low levels of physical activity and unhealthy dietary patterns, which are prevalent among adolescents worldwide and in New Zealand,^4^ are key risk factors.^5^

About four in five adolescents globally do not meet the internationally recommended guideline of 60 min of moderate-to-vigorous intensity physical activity per day.^6^ Active transport to and from school, solely or combined with motorised transport, provides an opportunity for adolescents to engage in physical activity and increase the likelihood of meeting international guidelines,^7^ especially among adolescents with low levels of physical activity.^8^ However, travel through an obesogenic environment^9^ (i.e. an environment that discourages physical activity and promotes unhealthy food choices) to or from school may facilitate adolescents to purchase and consume unhealthy foods or drinks.^10^ These foods typically offer little nutritional value yet are high in calories, saturated fats, trans fats, sugars and sodium and are often referred to as unhealthy snacks or ‘junk food’.^11^ Generally, snacking refers to the consumption of small amounts or portions of food or drinks between regular mealtimes.^12^ Snacking can occur at any time during the day, but after school has been reported to be the most common period for adolescents.^13^ Snacking can significantly contribute to daily total energy intake. For example, a Chilean study reported that on average adolescents had 2.3 servings of unhealthy snack food per day which constituted 27.4% of their daily energy intake.^14^

Although the school commute period can be a relatively small ‘window of time’, the environmental exposures en route to and from school may be associated with adolescents’ dietary behaviours,^15^ in addition to other influential individual and wider environmental factors such as socioeconomic deprivation.^16–18^ Unhealthy food outlets, such as fast-food and convenience stores are often present in school neighbourhoods,^16,19^ as well as along adolescents’ school route. Access to unhealthy food outlets is associated with unhealthy snacking in adolescents, particularly for those living in high-deprivation neighbourhoods.^20,21^ However, research on the purchase and consumption of unhealthy snacks or soft drinks among adolescents during the school journey is limited.^20,22^ For example, a Scottish study reported 42% of secondary school students sometimes purchased food or drinks on the way to or from school, but little else was reported.^20^ Previous studies have investigated the relationship between food and drink purchases, exposure to food outlets and school transport modes but these were in children^23,24^ and not adolescents. Compared with children, adolescents typically have more freedom to travel independently without supervision, and they have more disposable income which enables autonomous food choices.^15,25^ The aims of this study were: (1) to identify whether adolescents’ purchase and consumption of unhealthy snacks or soft drinks was associated with adolescents’ school transport mode and presence of food outlets along the estimated home-to-school route and (2) to examine whether food outlet density on adolescents’ estimated school routes differed between active, motorised and mixed transport users.

## Materials and Methods

### Study setting and participants

This study is part of the Built Environment and Active Transport to School Natural Experiment (BEATS-NE),^26^ a cross-sectional survey conducted in all 12 secondary schools in Dunedin, New Zealand, in 2020–2022. Dunedin is the seventh largest city by population and second largest city by territorial land area in New Zealand and is located in the Otago region. Data collection was completed between March 2020 and June 2022 during which there were two interruptions due to COVID-19 pandemic lockdown restrictions imposed in New Zealand. Data were collected only during the periods when schools were open. Ethics approval for this study was obtained from the University of Otago Human Research Ethics Committee (reference: 17/188; 14 December 2017) and Auckland University of Technology Ethics Committee (21/314; 24 September 2021). The study was registered in the Australian New Zealand Clinical Trial Registry (ANZCTR); trial registration number (ACTRN12619001335189).

**Figure 1:**
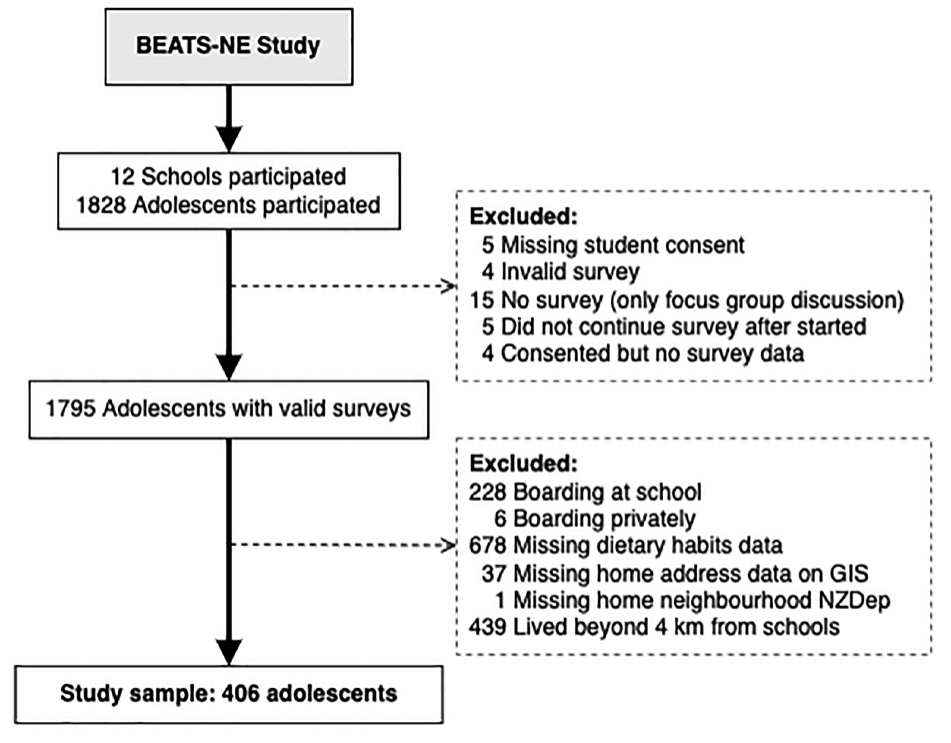
Flow chart of study participants included in this analysis Notes: GIS: Geographical Information System. NZDep: New Zealand Index of Deprivation.

Following the BEATS-NE Study methodology detailed elsewhere,^26^ adolescents aged 13 to 18 years were recruited through their schools. They were given study information 2–4 weeks prior to data collection, and those who agreed to participate provided signed consent prior to the survey start. Parents were informed, but their consent was not required under our ethics approval and agreement with the schools. Among 1828 adolescents who participated in this study, those for whom a signed consent was not linked with survey data, and those who had an invalid survey, no survey data, missing dietary habits data, missing home address and neighbourhood deprivation data, boarded at school or boarded privately or lived beyond 4 km from school were excluded (Figure 1). Therefore, data from 406 adolescents were included in this analysis.

### Measures

#### Student survey

Participating adolescents completed a 30- to 40-min online survey during a school class, supervised by research staff. This analysis included survey items that related to demographic characteristics (age, gender and ethnicity), adolescents’ transport to and from school and unhealthy snacking behaviour during the school journey. Adolescents self-reported their home address which was used to obtain the home-to-school route and distance.

Adolescents reported school transport modes separately for journeys to and from school using 11 different transport mode options and five response categories (‘never’, ‘rarely’, ‘sometimes’, ‘most of the time’ and ‘all of the time’).^27^ For this analysis, the dominant school transport modes (used ‘most/all of the time’) and multimodal transport were used to classify adolescents into three categories: active transport (‘on foot’, ‘by bike’, ‘by eBike’ or ‘by eScooter’), motorised transport (‘by car (driven by others)’, ‘by car (driving myself)’, ‘by school bus’ or ‘by public transport’) and mixed transport (‘by bus and on foot’, ‘by car and on foot’ or ‘other modes or combinations’ when combined active and motorised transport modes were used).^27^

Adolescents self-reported the frequency of both the purchase and consumption of: (1) unhealthy snacks (e.g. sweets, chips or ice cream) and (2) soft drinks, energy drinks or fruit juice (collectively referred to as soft drinks from here on), on the way to and from school, separately. The unhealthy snacking behaviour was assessed using the question ‘How often do you usually . . .?’ with six response categories ranging from zero to five times per week.^28^

#### Environmental data

Environmental variables were calculated in ArcGIS version 10.8.1, between July and November 2022. Adolescents’ home and school addresses were geo-coded to determine home-to-school distance, to estimate the shortest school route and to calculate area-level home neighbourhood deprivation using the New Zealand Index of Deprivation (NZDep).^29^ NZDep is an area-based measure of level of deprivation in New Zealand for the smallest geographical area (meshblock). It uses 9 New Zealand Census variables and is presented as deciles with Decile 1 representing 10% of the least deprived areas in New Zealand and Decile 10 being the most deprived areas. For this analysis, home neighbourhood deprivation was categorised into three groups: low-deprivation (NZDep deciles 1-3), mid-deprivation (NZDep deciles 4-7) and high-deprivation (NZDep deciles 8-10).

##### Home-to-school distance and estimated school route

The home-to-school distance was calculated using a custom geoprocessing script using the origin-destination tool in ArcGIS Network Analyst based on the walkable street network from each adolescent’s home address to their school. The estimated shortest route from each adolescent’s home address to their school’s main entrance was used in this study. A 4-km cut-off distance from home to school was used to ensure a balanced comparison between the three different school transport mode categories as very few adolescents walk or cycle to school if living beyond 4 km from their school.^30^ In this study, only 1% of those who lived more than 4 km from their school used active school transport, which is similar to adolescents’ transport to school patterns across the Otago region, New Zealand.^31^

##### Food outlets

Food outlet data within the Dunedin City Council boundary were generated using the Google Maps Platform ‘place types’ function that supports place searches in Google Places Application Programming Interface (API). Two approaches were used to retrieve food outlet data: (1) the selection of food outlet categories from a predetermined list of 96 ‘place types’ and (2) a free text search where Google matches specified text with the places’ name, description and reviews. The Google retrieved list of food outlets in Dunedin was audited using a modified virtual ground-truth method,^32^ where the validity of each food outlet was confirmed using Google Maps, Google Street View, the food outlet’s webpage (if it existed) or Facebook page, phone calls to the food outlet, local knowledge or visits to the address listed for the food outlet when all other approaches yielded unclear results. Validity was defined as an outlet having food or drink sales at the premise and operating during the time of the student survey. Each valid food outlet was categorised into one of eight food outlet types: bakery, café, convenience store, fast-food outlet, fresh-food store, restaurant, supermarket or takeaway. The definitions of the eight food outlet types are presented in Supplement 1. The count of each food outlet type along the estimated home-to-school route for each adolescent was calculated in geographic information system (GIS) using a custom geoprocessing script with the origin–destination tool. Three food outlet variables were derived for this analysis: (1) variety: count of each different food outlet type, (2) availability: binary option of presence or absence of food outlets along the estimated home-to-school route and (3) count of food outlets per kilometre: sum of the number of all food outlets along the estimated route to school divided by each adolescent’s estimated home-to-school distance (in km).

### Statistical analysis

Sociodemographic characteristics were analysed using descriptive statistics. Continuous data were reported as mean ± standard deviation (SD) and categorical data as numbers and frequencies (%). Differences between categorical variables were compared using the chi-square test. Schools are strata in the structure of the data, and this had a negligible effect on the estimated coefficients and their standard errors. Binary logistic regression models were fitted to estimate the odds of purchasing and consuming unhealthy snacks or soft drinks on both the journey to and from school, with explanatory variables being school transport modes, home-to-school distance, home neighbourhood deprivation and total food outlet counts per kilometre of the estimated home-to-school route. Individual-level covariates in the regression model were age, gender and ethnicity. A *p*-value of < 0.05 was considered statistically significant. Analyses were performed using SPSS software version 29.0.

## Results

The mean age of the 406 adolescents was 15.1 ± 1.4 years, 50.7% were boys, 63.5% were New Zealand European, and one-quarter lived in a high-deprivation neighbourhood (Table 1). The median home-to-school distance was 1.9 km, and active transport was used by 37.9% and 42.7% of adolescents to get to, and from, school, respectively.

**Table 1:** Sociodemographic and travel characteristics of study participants.

		All participants (*n* = 406)
		*N* (%)
Age (mean ± SD; years)		15.1 ± 1.4
Gender	Male	206 (50.7%)
	Female	194 (47.8%)
	Gender diverse	6 (1.5%)
Ethnicity	New Zealand European	258 (63.5%)
	Ma-ori	60 (14.8%)
	Pacific Islands	16 (3.9%)
	Asian	22 (5.4%)
	Other	50 (12.3%)
Distance to school (median (IQR); km)		1.9 (1.3–2.9)
Home neighbourhood deprivation	Low	153 (37.7%)
	Mid	151 (37.2%)
	High	102 (25.1%)
Transport TO school^a^	Active	150 (37.9%)
	Motorised	162 (40.9%)
	Mixed	84 (21.2%)
Transport FROM school^b^	Active	164 (42.7%)
	Motorised	144 (37.5%)
	Mixed	76 (19.8%)

IQR: interquartile range.

a Transport to school data were missing for 10 adolescents.

b Transport from school data were missing for 22 adolescents.

### Purchase and consumption of unhealthy snacks or soft drinks during the school journey

Overall, 26.4% of adolescents purchased and consumed unhealthy snacks or soft drinks on the way to school, and 41.4% on the way home from school at least 1 day per school week. The proportion of adolescents purchasing and consuming unhealthy snacks or soft drinks during the school journey differed by their transport modes both to school (*p* = 0.002) and from school (*p* < 0.001) with mixed transport users having the highest proportion who purchased and consumed unhealthy snacks (Table 2). No significant difference was found in unhealthy snacking behaviour by home-to-school distance or home neighbourhood deprivation. The proportion of adolescents who purchased and consumed unhealthy snacks during the school journey differed by total food outlet counts per kilometre of the estimated home-to-school route, *p* = 0.021, but not by availability or variety of food outlets.

**Table 2:** The proportion of adolescents who did or did not purchase and consume unhealthy snacks or soft drinks during their school journey by school transport modes, home-to-school distance, home neighbourhood deprivation and food outlet variables.

Variables	Groups	Proportion of adolescents who purchased and consumed unhealthy snacks or soft drinks					
		On the way TO school		*p-*value	On the way FROM school		*p*-value
		Never	At least 1 day/school week		Never	At least 1 day/school week	
		*N* (%)	*N* (%)		*N* (%)	*N* (%)	
All adolescents		299 (73.6%)	107 (26.4%)		238 (58.6%)	168 (41.4%)	
School transport modes	Active	117 (78.0%)	33 (22.0%)	0.002	105 (64.0%)	59 (36.0%)	<0.001
	Motorised	128 (79.0%)	34 (21.0%)		93 (64.6%)	51 (35.4%)	
	Mixed	50 (59.5%)	34 (40.5%)		27 (35.5%)	49 (64.5%)	
Home-to-school distance	⩽2.25 km	170 (72.6%)	64 (27.4%)	0.595	145 (62.0%)	89 (38.0%)	0.110
	>2.25 and ⩽4 km	129 (75.0%)	43 (25.0%)		93 (54.1%)	79 (45.9%)	
Home neighbourhood deprivation	Low	118 (77.1%)	35 (22.9%)	0.103	99 (64.7%)	54 (35.3%)	0.114
	Mid	114 (75.5%)	37 (24.5%)		80 (53.0%)	71 (47.0%)	
	High	67 (65.7%)	35 (34.3%)		59 (57.8%)	43 (42.2%)	
*Food outlets on the estimated school route*							
Variety (number of different food outlet types)^a^	0	133 (73.1%)	49 (26.9%)		116 (63.7%)	66 (36.3%)	
	1 to 3	86 (71.1%)	35 (28.9%)	0.522	67 (55.4%)	54 (44.6%)	0.161
	4 to 7	80 (77.7%)	23 (22.3%)		55 (53.4%)	48 (46.6%)	
At least one food outlet of any type	No	133 (73.1%)	49 (26.9%)	0.815	116 (63.7%)	66 (36.3%)	0.059
	Yes	166 (74.1%)	58 (25.9%)		122 (54.5%)	102 (45.5%)	
Total food outlet count per kilometre	None	133 (73.1%)	49 (26.9%)		116 (63.7%)	66 (36.3%)	
	Up to 1	32 (61.5%)	20 (38.5%)	0.062	22 (42.3%)	30 (57.7%)	0.021
	More than 1	134 (77.9%)	38 (22.1%)		100 (58.1%)	72 (41.9%)	

a Food outlet types: bakery, café, convenience store, fast-food outlet, fresh-food store, restaurant, supermarket or takeaway.

### The odds of adolescents purchasing and consuming unhealthy snacks or soft drinks during the school journey

In the adjusted regression models, adolescents’ purchase and consumption of unhealthy snacks or soft drinks was associated with school transport modes, but not with the total food outlet counts per kilometre of the estimated home-to-school route (Table 3). Adolescents using mixed transport modes, compared with active transport, had significantly higher odds of purchasing and consuming unhealthy snacks or soft drinks both on the way to and from school. Home neighbourhood deprivation was also associated with adolescents’ unhealthy snacking behaviour on the way from school, but not to school. Compared to adolescents who lived in low-deprivation neighbourhoods, those who lived in mid-deprivation neighbourhoods had higher odds of unhealthy snacking on the way from school.

**Table 3:** Adjusted odds ratios (ORs) of adolescents purchasing and consuming unhealthy snacks or soft drinks during school travel.

		Odds of adolescents purchasing and consuming unhealthy snacks or soft drinks			
		On the way TO school		On the way FROM school	
		OR (95% CI)	*p*-value	OR (95% CI)	*p*-value
School transport modes	Active *(ref)*	1.00		1.00	
	Motorised	1.09 (0.60, 1.99)	0.782	0.91 (0.53, 1.56)	0.722
	Mixed	2.77 (1.41, 5.44)	0.003	2.69 (1.41, 5.16)	0.003
Home-to-school distance	⩽2.25 km *(ref)*	1.00		1.00	
	>2.25 km and ⩽4 km	0.67 (0.37, 1.21)	0.182	1.14 (0.66, 1.95)	0.649
Home neighbourhood deprivation	Low *(ref)*	1.00		1.00	
	Mid	1.08 (0.61, 1.92)	0.794	1.93 (1.16, 3.19)	0.011
	High	1.51 (0.81, 2.81)	0.194	1.32 (0.74, 2.33)	0.348
Total food outlet count per kilometre of school route	None *(ref)*	1.00		1.00	
	Up to 1	1.28 (0.57, 2.88)	0.552	1.47 (0.69, 3.17)	0.320
	More than 1	0.66 (0.37, 1.16)	0.149	1.00 (0.61, 1.64)	0.999

### Food outlets on adolescents’ estimated home-to-school routes

Overall, 44.8% of adolescents did not have any food outlets on their estimated home-to-school route while 46.8% had two or more different food outlet types. The total number of food outlets on adolescents’ estimated home-to-school routes varied widely ranging from none to 62. On average, there were 1.54 food outlets for every 1 km of adolescents’ estimated home-to-school route. Restaurants and takeaways represented more than half of the total food outlet counts, whereas fresh-food stores were uncommon (3%). The proportion of adolescents with no, up to one or more than one food outlet(s) per kilometre of their estimated home-to-school route differed significantly by school transport mode (Table 4).

**Table 4:** The proportion of adolescents with no, up to one or more than one food outlet per kilometre of their estimated home-to-school route by usual school transport mode category.

	Usual transport modes to school			
	Active *(N* *=* *150)*	Motorised *(N* *=* *162)*	Mixed *(N* *=* *84)*	*p*-value
	*N* (%)	*N* (%)	*N* (%)	
Total food outlet count per kilometre of estimated school route				
None	82 (54.7%)	67 (41.4%)	29 (34.5%)	
Up to 1	10 (6.7%)	26 (16.0%)	16 (19.0%)	0.006
More than 1	58 (38.7%)	69 (42.6%)	39 (46.4%)	

## Discussion

This study examined adolescents’ purchase and consumption of unhealthy snacks and soft drinks during their school journey and the association with school transport modes and food outlets on the estimated home-to-school routes among adolescents who lived less than 4 km from their school. The key findings were (1) 26.4% of adolescents purchased and consumed unhealthy snacks or soft drinks on the way to school and 41.4% on the way from school at least once during the school week; (2) mixed transport users and adolescents residing in mid-deprivation neighbourhoods had higher odds of unhealthy snacking than active transport users and those residing in low-deprivation neighbourhoods; (3) the proportion of adolescents who purchased and consumed unhealthy snacks or soft drinks during the school journey differed by total food outlet counts per kilometre of the estimated home-to-school route, but no significant associations were found in the adjusted model; (4) 44.8% of adolescents did not have any food outlets on their estimated home-to-school route; and (5) the proportion of adolescents who had no, up to one or more than one total food outlet counts per kilometre of the estimated home-to-school route differed by their usual transport modes to school. Taken together, these findings suggest that both adolescents’ unhealthy snacking behaviour during the school journey and total food outlet counts per kilometre of estimated home-to-school routes were associated with their school transport modes.

Overall, there was a higher proportion of adolescents who purchased and consumed unhealthy snacks or soft drinks on the way from, than to school, which was also reported in a previous similar study among adolescents residing in small to medium urban and rural settlements across the Otago region, New Zealand,^28^ as well as in a large Australian study.^13^ Possible explanations for this finding are that this time of day may correspond to physiological needs due to adolescence being a period of rapid growth and development,^33^ and it can be a time of day when adolescents have time to socialise with their friends and to visit food outlets, while being independent of adult supervision.^16^ There were few fresh-food stores on the estimated home-to-school routes, which suggests that the food outlets that adolescents encounter and the type of food and drinks available along their school journey are more likely to be unhealthy than healthy.^19,34^ Moreover, adolescents who consume food while ‘on the journey’ compared to eating at home or at school are more likely to eat unhealthy snacks or soft drinks than healthy food choices such as fruit.^35^

This study observed that unhealthy snacking behaviour on the school journey was more common among mixed transport users than those using active or motorised transport, which is a novel and unexpected finding. Our observations may be associated with convenience and time constraints among users of different transport modes. In a Canadian study, among adolescents aged 9–13 years, the odds of purchasing junk food adjusted for exposure to food outlets during the school commute was higher among those who travelled by car than those who used active travel.^23^ The Canadian study did not include mixed transport users, who are likely to have higher levels of autonomy during their school journey compared to adolescents who use motorised transport only to travel to and from school. Having some autonomy may facilitate unhealthy snacking behaviours.^36^ In addition, it is likely that mixed transport users live further from school and therefore take longer to get home than active transport users and this may increase the likelihood of hunger and prompt adolescents to stop for food.^22^

The association between unhealthy snacking and school transport modes may also be partly explained by the availability and variety of food outlets along the school route. Mixed transport users had the highest proportion of adolescents with more than one food outlet per kilometre of their estimated home-to-school route than active and motorised transport users. This implies there were more opportunities for mixed transport users to purchase and consume food during their school journey. While this study did not explore what portion of the school journey was active and what portion was motorised, it is likely that mixed transport users had to wait for their public transport or to be picked up, particularly in the central city area.^16^ It is worth noting that public transport hubs have been observed to encourage unhealthy food choices as they are often located next to food outlets,^37^ which could potentially influence adolescents’ decisions to purchase and consume unhealthy food and drinks.^37^ The period of travel to and from school, particularly when using active or public transport as part of the journey, is typically independent of adult supervision, thereby allowing adolescents to access food outlets^15^ and freedom of choice to spend money on unhealthy snacks or soft drinks.^25^ The perceived lack of importance placed on healthy options,^10^ coupled with the affordability of unhealthy food choices may also encourage unhealthy snacking.^16^

There was a lack of association between adolescents’ unhealthy snacking and the total food outlet counts per kilometre of the estimated home-to-school route in the adjusted regression model. A possible explanation for this result could be that almost half of the adolescents did not have any food outlets on their estimated home-to-school route, which may have biased the estimation. Another possible explanation could be that food outlet exposure is not related to unhealthy snacking behaviour^17^ or that other behavioural or environmental factors may mediate this relationship.^12^ For example, this study observed significantly higher odds of unhealthy snacking among adolescents who lived in mid- but not high-deprivation neighbourhoods than those who lived in low-deprivation neighbourhoods in the adjusted model. This finding is inconsistent with other studies and a previous similar study in 2018 among adolescents residing in small to medium urban and rural schools in Otago, New Zealand.^28^ The association between unhealthy snacking and living in mid-deprivation neighbourhoods may be attributed to increasing poverty and less disposal income to spend on discretionary items like unhealthy snacks among low-income families, particularly since the onset of COVID-19 pandemic.^38^

### Implications

Although active transport may increase physical activity levels in adolescents and provide health benefits,^7^ the combination of active and motorised transport, that is mixed transport, was positively associated with the purchase and consumption of unhealthy snacks or soft drinks during the school journey. Mixed transport users also had a higher availability of food outlets on their estimated home-to-school route, which is likely to have facilitated unhealthy snacking.^18^ It is necessary to limit access to unhealthy food during the school journey, as it has the potential to obscure adolescents’ ability to distinguish between healthy and unhealthy nutrition.^34^ In addition, while beyond the scope of this study, food environments at adolescents’ places of activity, including schools, should also be considered along with other influential factors such as food marketing and food prices when planning health promotion activities to encourage healthy eating among adolescents.^39^

### Study strengths and limitations

The strengths of this study include participation of adolescents from all 12 secondary schools in the study city, rigorous ground-truthing of Google retrieved food outlet data, the inclusion of mixed transport to school as a separate category (in addition to active and motorised transport), and the combination of both purchase and consumption of unhealthy snacks or soft drinks on the way both to and from school. The limitations of the study include the use of self-reported unhealthy snacking behaviour and the use of estimated routes from home to school rather than actual routes. Self-reported survey data are prone to response bias but remains widely used as a non-invasive method to evaluate food intake in young people.^35^ GIS estimated shortest home-to-school routes are likely to be different from the actual routes taken by adolescents particularly after school.^40^ In addition, the portions of the school journey travelled using the different modes among mixed transport users were not collected. Further research efforts should use adolescents’ actual school routes travelled, collect data on the portion of the school journey travelled using different transport modes and collect more comprehensive dietary behaviour data.

## Conclusion

This study provides insights into snacking behaviours during adolescents’ journey to and from school and the food outlets adolescents may be exposed to on their school route. School transport modes were associated with both adolescents’ unhealthy snacking during the school journey and the presence of food outlets on the estimated home-to-school route. The school commute period may represent only a small ‘window of time’ during the day, but how adolescents get to and from school and the routes they take may influence adolescents’ unhealthy snacking behaviour. Policy measures which minimise exposure to unhealthy food outlets (e.g. zoning regulations for food outlets particularly around schools) may reduce unhealthy snacking behaviour in adolescents, and thereby contribute to reducing the prevalence of obesity and its health effects.

## Supplemental Material

sj-docx-1-rsh-10.1177_17579139241257091 – Supplemental material for Adolescents’ unhealthy snacking behaviour during the school journey and the association with transport modes and food outlets along the school route Supplemental material, sj-docx-1-rsh-10.1177_17579139241257091 for Adolescents’ unhealthy snacking behaviour during the school journey and the association with transport modes and food outlets along the school route by ML Situmorang, S Mandic, M Smith, M Keall, N Donnellan and KJ Coppell in Perspectives in Public Health
